# Evidence for both sequential mutations and recombination in the evolution of *kdr* alleles in *Aedes aegypti*

**DOI:** 10.1371/journal.pntd.0008154

**Published:** 2020-04-17

**Authors:** Yinjun Fan, Patrick O'Grady, Melissa Yoshimizu, Alongkot Ponlawat, Phillip E. Kaufman, Jeffrey G. Scott

**Affiliations:** 1 Department of Entomology, Comstock Hall, Cornell University, Ithaca, New York, United States of America; 2 Vector-Borne Disease Section, Division of Communicable Disease Control, Center for Infectious Diseases, California Department of Public Health, Sacramento, California, United States of America; 3 Department of Entomology, USAMD-AFRIMS, Bangkok, Thailand; 4 Entomology and Nematology Department, University of Florida, Gainesville, Florida, United States of America; International Centre for Genetic Engineering and Biotechnology, INDIA

## Abstract

**Background:**

*Aedes aegypti* is a globally distributed vector of human diseases including dengue, yellow fever, chikungunya, and Zika. Pyrethroid insecticides are the primary means of controlling adult *A*. *aegypti* populations to suppress arbovirus outbreaks, but resistance to pyrethroid insecticides has become a global problem. Mutations in the *voltage-sensitive sodium channel (Vssc)* gene are a major mechanism of pyrethroid resistance in *A*. *aegypti*. *Vssc* resistance alleles in *A*. *aegypti* commonly have more than one mutation. However, our understanding of the evolutionary dynamics of how alleles with multiple mutations arose is poorly understood.

**Methodology/Principal findings:**

We examined the geographic distribution and association between the common *Vssc* mutations (V410L, S989P, V1016G/I and F1534C) in *A*. *aegypti* by analyzing the relevant *Vssc* fragments in 25 collections, mainly from Asia and the Americas. Our results showed all 11 Asian populations had two types of resistance alleles: 1534C and 989P+1016G. The 1534C allele was more common with frequencies ranging from 0.31 to 0.88, while the 989P+1016G frequency ranged from 0.13 to 0.50. Four distinct alleles (410L, 1534C, 410L+1534C and 410L+1016I+1534C) were detected in populations from the Americas. The most common was 410L+1016I+1534C with frequencies ranging from 0.50 to 1.00, followed by 1534C with frequencies ranging from 0.13 to 0.50. Our phylogenetic analysis of *Vssc* supported multiple independent origins of the F1534C mutation. Our results indicated the 410L+1534C allele may have arisen by addition of the V410L mutation to the 1534C allele, or by a crossover event. The 410L+1016I+1534C allele was the result of one or two mutational steps from a 1534C background.

**Conclusions/Significance:**

Our data corroborated previous geographic distributions of resistance mutations and provided evidence for both recombination and sequential accumulation of mutations contributing to the molecular evolution of resistance alleles in *A*. *aegypti*.

## Author summary

Mutations in the *voltage-sensitive sodium channel* (*Vssc*) gene in *Aedes aegypti* can confer resistance to insecticides and threaten vector control programs. To date, twelve *Vssc* mutations have been identified in pyrethroid resistant *A*. *aegypti* populations, and F1534C, V1016G/I, S989P and V410L commonly are found alone or in combination. However, the evolutionary dynamics of how alleles came to have multiple mutations is less known. In this study, we found 1534C and 989P+1016G alleles were widely distributed in Asian populations. In contrast, the distinct 410L, 410L+1534C, 410L+1016I+1534C and 1534C alleles were distributed in populations from the Americas. The 410L+1534C allele appears to have arisen by accumulation of the 410L mutation in an individual already having the 1534C allele or by a crossover event. The 410L+1016I+1534C arose by additional mutations from 1534C background. Our data corroborates previous geographic distributions of resistance mutations and provides evidence for both recombination and sequential accumulation of mutations contributing to the molecular evolution of resistance mutations in *A*. *aegypti*.

## Introduction

*Aedes aegypti* is a vector of four important human disease viruses: dengue, yellow fever, chikungunya, and Zika [1]. Pyrethroids are the most common class of insecticides used to control adult *A*. *aegypti*. The decades-long intensive use of pyrethroid insecticides has resulted in the evolution of resistance in *A*. *aegypti* across the globe [2]. The voltage-sensitive sodium channel (VSSC) is the target site of pyrethroid insecticides, and mutations in *Vssc* are a major mechanism of pyrethroid resistance [3,4]. These resistance conferring mutations are collectively known as *knockdown resistance* (*kdr*).

To date, twelve *Vssc* mutations have been identified in pyrethroid resistant *A*. *aegypti* populations (Table 1). The G923V+I1011M, L982W, I1011M and V1016G mutations were first identified in 2003 [5]. I1011V and V1016I were identified in 2007 [6]. V1016G+D1763Y was identified in 2009 [7]. S989P+V1016G and F1534C were identified in 2010 [8,9]. V1016G+F1534C and S989P+V1016G+F1534C were identified in 2014 [10]. T1520I+F1534C was identified in 2015 [11]. V410L+F1534C was identified in 2017 [12]. V410L, V410L+F1534C, V410L+V1016I and V410L+V1016I+F1534C were identified in 2018 [13,14]. V1011M+V1016G was identified in 2019 [15]. Amino acid positions of these mutations are numbered based on the house fly VSSC (Genbank accession number: AAB47604). Of the known *kdr* mutations, F1534C, V1016G/I, S989P and V410L are common (Table 1). The F1534C mutation has been found in 58 out of 89 studies of pyrethroid resistant *A*. *aegypti* populations globally, while S989P (13 of 49 detections), V1016G (29 of 58 detections) and V1016I (33 of 89 detections) vary geographically [2,16,17]. To date, S989P has been found only in Asia [2,18,19]. V1016G mutations have been found in Asia [2,18,19] and the Americas [15]. In contrast, the V1016I mutation has been detected only in the Americas, Europe and Africa [2,16,17,20]. In 2017, the V410L mutation was first found in *A*. *aegypti* from Brazil [12]. Subsequently, the V410L mutation was detected in Colombian and Mexican populations [13,14]. Given the history of the discovery of the different mutations, several studies have only reported on a subset of the mutations that are now known, giving an incomplete picture of the distribution of each mutation and of the alleles present (Table 1). For example, several studies have focused on the V1016I mutation, without considering the V410L and F1534C mutations [6,21–23], even though the V410L+V1016I+F1534C allele can be common [13,14]. Such an incomplete listing of mutations makes understanding the evolution of resistance alleles particularly challenging [4].

**Table 1:** *Vssc* mutations detected in pyrethroid resistant *A*. *aegypti* populations.

Allele	Mutation*											Continents	Country	Reference
	V410L	G923V	L982W	S989P	I1011M	I1011V	V1016I	V1016G	T1520I	F1534C	D1763Y			
V410L	+						-			-		America	Colombia	[13]
	+						-			-			Mexico	[14]
L982W		-	+	-	-	-	-	-				Asia	Vietnam	[5]
I1011V					-	+	-	-				America	Multiple Latin American	[6]
					-	+	-	-				Asia	Thailand	[59]
I1011M			-	-	+	-	-	-				America	Brazil	[55]
V1016I					-	-	+	-				America	Multiple Latin American	[6]
							+						Colombia	[21]
							+			-			Colombia	[45]
							+						Mexico	[22]
							+			-			Mexico	[58,60]
	-						+			-			Mexico	[14]
							+			-			Grand Cayman	[61]
							+			-			Brazil	[62–64]
							+			-			Venezuela	[65]
			-	-	-	-	+	-					USA	[23]
							+			-			USA	[44]
							+			-			Colombian Caribbean	[66]
V1016G		-	-	-	-	-	-	+				Asia	Thailand	[5]
		-	-	-	-	-	-	+					Indonesia	[5]
				-				+		-			Indonesia	[67]
								+		-			Indonesia	[19]
					-	-	-	+		-			Vietnam	[68]
				-	-	-	-	+		-			Malaysia	[69]
				-				+					Malaysia	[70]
					-	-	-	+		-	-		Singapore	[71]
				-	-	-	-	+		-	-		Taiwan, China	[17,56]
				-	-	-	-	+		-			India	[72]
F1534C					-	-	-	-		+		Asia	Vietnam	[68]
	-	-	-	-	-	-	-	-	-	+			Thailand	[9]
				-	-	-	-	-		+	-		Singapore	[36]
				-	-	-	-	-		-	-		Singapore	[71]
				-	-	-	-	-		+			Myanmar	[10]
				-				-		+			Indonesia	[67]
								-		+			Indonesia	[19]
				-	-	-	-	-		+			Indonesia	[73]
				-	-	-	-	-		+			Malaysia	[69]
				-				-		+			Malaysia	[38]
										+			India	[74]
				-	-	-	-	-		+			India	[72]
				-				-		+			China	[75]
				-	-	-	-	-		+	-		Taiwan, China	[17,56]
							-			+		America	Venezuela	[65]
	-	-	-	-	-	-	-	-	-	+	-		Brazil	[12]
							-			+			Brazil	[62–64]
							-			+			Mexico	[58,60,76]
	-						-			+			Mexico	[14]
							-			+			Jamaica	[77]
	-						-			+			Colombia	[13]
							-			+			Colombia	[45]
							-			+			Puerto Rico	[46]
							-			+			USA	[44]
				-	-					+	-		USA	[78]
							-			+			Colombian Caribbean	[66]
				-	-	-	-	-		+		Africa	Ghana	[17]
							-			+			Burkina Faso	[20]
				-			-	-		+			Burkina Faso	[79]
							-			+		Europe	Madeira Island	[16]
V410L+V1016I	+						+			-		America	Colombia	[13]
	+						+			-			Mexico	[14]
V410L+F1534C	+	-	-	-	-	-	-	-	-	+	-	America	Brazil	[12]
	+						-			+			Colombia	[13]
	+						-			+			Mexico	[14]
V410L+V1016I+F1534C	+						+			+		America	Colombia	[13]
	+						+			+			Mexico	[14]
G923V+I1011M		+	-	-	+	-	-	-				America	French Guyana	[5]
		+	-	-	+	-	-	-					Brazil	[5,55]
		+	-	-	+	-	-	-					Martinique	[5]
S989P+V1016G		-	-	+	-	-	-	+				Asia	Thailand	[8,80]
	-	-	-	+	-	-	-	+	-	-	-		Singapore	[36]
				+	-	-	-	+		-			Myanmar	[10]
				+				+		-			China	[75]
				+	-	-	-	+		-	-		Taiwan, China	[17,56]
				+				+		-			Indonesia	[67]
				+	-	-	-	+		-			Saudi Arabia	[18]
				+				+		-			Sri Lanka	[81]
				+				+					Malaysia	[70]
				+				+		-			Malaysia	[38]
S989P+V1016G+F1534C				+	-	-	-	+		+		Asia	Myanmar	[10]
				+	-	-	-	+		+			Saudi Arabia	[18]
				+				+		+			Malaysia	[38]
V1011M+V1016G				-	+	-	-	+		-		America	Panama	[15]
V1016I+F1534C							+			+		America	Grand Cayman	[61]
					-	-	+	-		+			Brazil	[82]
							+			+			Brazil	[62–64]
							+			+			Venezuela	[65]
							+			+			Mexico	[58,60,76]
	-						+			+			Mexico	[14]
							+			+			Mexico	[58]
							+			+			Jamaica	[77]
	-						+			+			Colombia	[13]
							+			+			Colombia	[45]
							+			+			Puerto Rico	[46]
							+			+			USA	[44]
							+			+			Colombian Caribbean	[66]
				-	-	-	+	-		+		Africa	Ghana	[17]
							+			+			Burkina Faso	[20]
				-			+	-		+			Burkina Faso	[79]
							+			+		Europe	Madeira Island	[16]
V1016G+F1534C					-	-	-	+		+	-	Asia	Singapore	[71]
				-	-	-	-	+		+			Myanmar	[10]
				-	-	-	-	+		+			India	[72]
V1016G+D1763Y	-	-	-	-	-	-	-	+	-	-	+	Asia	Taiwan, China	[7]
				-	-	-	-	+		-	+		Taiwan, China	[17,56]
T1520I+F1534C									+	+		Asia	India	[11]
Total^	9/18	3/12	1/14	13/49	5/44	2/43	33/89	29/58	1/6	58/89	2/13			

*A1007G mutation [47] was not included owing to the unclear role of this allele.

+ indicates the mutation was found.

- indicates the mutation was investigated, but it did not exist.

A black box indicates the mutation was not investigated.

^ The number of studies that found the mutation/the number of studies that examined the mutation.

Different studies have attempted to reconstruct the evolution of *kdr* alleles in *A*. *aegypti*. Saavedra-Rodriguez et al [14] examined three mutations (V410L, V1016I and F1534C) in populations of *A*. *aegypti* collected in Mexico from 2002 to 2012 and proposed that each arose independently and the 410L+1016I+1534C allele arose by two subsequent recombination events. A recent paper [4] proposed sequential evolution of the 989P+1016G+1534C allele, in which the 1016G allele was selected first, followed by 989P +1016G, and finally 989P+1016G+1534C. The 989P+1016G+1534C allele may have arisen from a crossing-over event [24], although sequential accumulation of mutations may also have played a role. The goal of the current study is to improve the understanding of how multiple mutations in *Vssc* have arisen and evolved. We examined nine *Vssc* mutations (V410L, L982W, S989P, A1007G, I1011V/M, V1016G/I and F1534C) and pattern types (see Materials and methods) of individuals from various geographical areas to investigate whether or not *Vssc* alleles with multiple mutations occur by (a) sequential accumulation of individual mutations, (b) by recombination between individual alleles or (c) some combination of the two mechanisms. Our results indicate the 410L+1534C allele could have evolved by both recombination and by addition of a second mutation to an existing resistance allele. This information will help to inform the evolution of *kdr* resistance in this important vector of human diseases.

## Materials and methods

### Rationale

**Fig 1:**
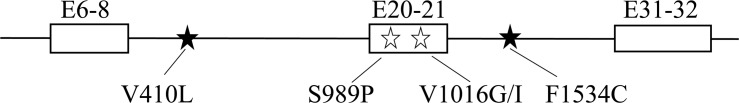
Diagram of *A*. *aegypti Vssc*. Regions that were sequenced are shown as boxes. Two mutations determined by ASPCR are shown as black stars. Stars indicate positions of five *kdr* mutations that may confer resistance. Diagram is not to scale. The L982W, A1007G and I1011M/V mutations (in the E20-21 region) are not shown as they were not found in any mosquitoes we tested.

*Vssc* in *A*. *aegypti* is >450 kb in length and contains >30 exons [25]. To investigate the evolution of *kdr* mutations in *Vssc*, we needed to explore the three regions containing mutations (V410L, S989P ± V1016G/I and F1534C) that are common in *A*. *aegypti* (Table 1). In addition, we wanted to capture sequence information near the 5′ and 3′ ends of *Vssc* to maximize the chances of detecting recombination events. We selected exons 6–8 and 31–32 for this purpose because their introns/exons were of a size appropriate for reliable PCR amplification and sequencing. Preliminary PCR and sequencing in the V410L or F1534C regions showed very limited sequence diversity (based on sequencing PCR products of about 1000 bp), beyond the presence or absence of the resistance mutations. Thus, for these regions we simply identified the presence or absence of the resistance mutation by allele-specific PCR (ASPCR) (see below). The L982W, S989P, A1007G, I1011M/V and V1016G/I mutations were investigated by sequencing a PCR product spanning exons 20–21. Thus, for each individual mosquito we sequenced three regions of *Vssc* (exon 6–8, exon 20–21 and exon 31–32) and genotyped for V410L and F1534C by ASPCR, giving us data across *Vssc* and providing details about which of the known resistance mutations were present (Fig 1).

### *A. aegypti* collections

We evaluated the *Vssc* sequences of 25 populations of *A*. *aegypti*. Eleven of these were from Asia, thirteen were from the Americas and one was from Africa. Our goal was to identify the most common *Vssc* alleles in each population. At least eight individuals from each population were used, except for California populations (LA, SD, TE and MD) in which four individuals were used. The Costa Rica and Puerto Rico strains were provided by BEI Resources (https://www.beiresources.org/Home.aspx). Details about populations were shown in Table 2. Mosquitoes were reared at 27°C (± 1°C) with 70–80% relative humidity, and a photoperiod of 14 L:10 D. Females were blood fed using membrane-covered water-jacketed glass feeders with cow blood (Owasco Meat Co., Moravia, NY).

**Table 2:** Locations where *A*. *aegypti* were collected.

Name	Location	Field/lab	Collection Date	Reference
LVP	Africa: Sierra Leone	Lab	Between 1935 and 1938	[83]
SP and SP_0_	Asia: Singapore	Field	2009	[24]
NSK	Asia: Nakhon Ratchasima, Samutprakarn, Thailand	Lab/Field	Between 2006 and 2017	This paper
BKK	Asia: Bangkok, Thailand^*d*^	Field	2018	This paper
UBN	Asia: Ubon Ratchathani, Thailand^*d*^	Field	2018	This paper
SNI	Asia: Suratthani, Thailand^*d*^	Field	2018	This paper
CMI	Asia: Chiang Mai, Thailand^*d*^	Field	2018	This paper
KPP	Asia: Kamphaeng Phet, Thailand^*d*^	Field	2018	This paper
HN	Asia: Hainan, China^*f*^	Field	2017	This paper
DZ	Asia: Danzhou, China^*f*^	Field	2017	This paper
RL	Asia: Ruili, China^*f*^	Field	2017	This paper
XSBN	Asia: Xishuangbanna, China^*f*^	Field	2017	This paper
SMK	North America: USA	Lab	before 1993	[24]
OL	North America: Orlando, Florida, USA	Lab	1952	This paper
AT	North America: Augustine, Florida, USA^*c*^	Field	2016	This paper
LA	North America: Los Angeles, California, USA^*a*^	Field	2017	This paper
SD	North America: San Diego, California, USA^*b*^	Field	2017	This paper
TE	North America: Tulare, California, USA^*a*^	Field	2017	This paper
MD	North America: Merced, California, USA^*a*^	Field	2017	This paper
NO	North America: New Orleans, USA	Lab	~1990	[84]
RK	South America: Cuba	Lab	unknown	[83]
MN	South America: Acacias, Meta, Colombia^*e*^	Field	2017	This paper
LM	South America: La Mesa, Colombia	Lab	2016	This paper
PR	South America: San Juan, Puerto Rico	Lab	2012	This paper
CR	South America: Puntarenas, Costa Rica	Lab	2001	[85]

^*a*^Adult females were collected and tested.

^*b*^Adult males were collected and tested.

^*c*^Random sample from collection of ~1500 eggs.

^*d*^Larvae and pupae (~300–500 total per site) were collected from water storage containers inside and around houses. Emerging *A*. *aegypti* adults were placed in cages (30 x 30 x 30 cm) and raised under laboratory conditions. The 1^st^ generation offspring were used in this study.

^*e*^The second generation from a collection of >300 eggs.

^*f*^Larvae (15–32 per site) were collected. Emerging *A*. *aegypti* adults were placed in cages and raised under laboratory conditions. The 1^st^ generation offspring were used in this study.

### DNA extraction

Genomic DNA was isolated from three legs of individual mosquitoes from each population using an alkali extraction method as follows. The legs were placed in individual wells of a 96-well PCR plate (BioRad, Hercules, CA, USA) containing three 2.3-mm diameter zirconia/silica beads (BioSpec Products, Bartlesville, OK, USA) and 10 μL 0.2 M NaOH. The samples were homogenized on a vortex mixer for 2 min and then incubated at 70°C for 10 min. Then 10 μL of neutralization buffer (360 mM Tris-HCl, pH 7.5 and 10 mM EDTA) and 80 μl of ddH_2_O were added to each well.

### Genotyping

PCR was carried out using 1 μL gDNA, 12.5 μL GoTaq Green Master Mix 2x (Promega, Madison, WI, USA), 9.5 μL ddH_2_O, and 2 μL of 10 μM forward and reverse primer mix. The primers and thermocycler conditions are shown in Table 3. The genotypes for *Vssc* exons 6–8, 20–21 and 31–32 were obtained by Sanger sequencing. PCR products were treated with ExoSAP (5 μL PCR product/1 μL of 1 u/μL ExoSAP mix), and sequenced (6 μL ExoSAP treated PCR product, 1 μL primer, 11 μL ddH_2_O) at the Cornell University Biotechnology Resource Center. PCR products with ambiguous sequences were cloned using the pGEM-T Vector System (Promega). Positive colonies were confirmed to have the expected size insert by PCR with primers T7 and SP6 designed from the plasmid sequence, and the PCR products were directly sequenced using the primer T7. Sequences were confirmed by analyzing two or more clones.

**Table 3:** *Vssc* primers used for genotyping individual *A*. *aegypti*.

Region/Mutation	Primer	Sequence	Purpose
E6-8^a^ 522 bp	E6-F	GGGTAATCTCGCTGCATTGAG	PCR amplification and sequencing
	E8-R	ACCCGACGAGTTCCCACAAAGAG	PCR amplification
E20-21^a^ 614 bp	E20-F	GACAATGTGGATCGCTTCCC	PCR amplification
	E21-R	GCAATCTGGCTTGTTAACTTG	PCR amplification and sequencing
E31-32^b^ 1128 bp	E31-F	CTCGTTCTTAGCGATCTCATCG	PCR amplification
	E32-R	CAGACATCCGCCGATCGTGAAG	PCR amplification
	E31-F1	CAAGAGCGGGCTGGACGATGTG	sequencing
V410L^a^	AaSCF9	TATCTGCCTTTCGTCTAATGACCC	PCR amplification
	AaSCR10	TTCCTCGGCGGCCTCTTC	PCR amplification
	410com-F	TATCTGCCTTTCGTCTAATGACCC	Allele-specific common primer
	V410-R	GACAATGGCCAAGATCAAATTGAC	Allele-specific susceptible genotype
	410L-R	GACAATGGCCAAGATCAAATTAAA	Allele-specific resistance genotype
F1534C*^c^	1534com-F	GGAGAACTACACGTGGGAGAAC	Allele-specific common primer and PCR amplification
	1534com-R	CGCCACTGAAATTGAGAATAGC	Allele-nonspecific outer primer and PCR amplification and sequencing
	F1534-R	GCGTGAAGAACGACCCGA	Allele-specific susceptible genotype
	1534C-R	GCGTGAAGAACGACCCGC	Allele-specific resistance genotype

^a^ Thermalcycler conditions: 94°C for 3 min, 35 × (94°C for 30 s, 55°C for 30 s, 72°C for 30 s) and 72°C for 10 min.

^b^ Thermalcycler conditions: 94°C for 3 min, 35 × (94°C for 30 s, 55°C for 30 s, 72°C for 1min) and 72°C for 10 min.

^c^ Thermalcycler conditions: 94°C for 3 min, 35 × (94°C for 30 s, 60°C for 30 s, 72°C for 30 s) and 72°C for 10 min.

* The methods were previously reported [75]

ASPCR was used to determine the V410L and F1534C genotypes. Each ASPCR was evaluated on a 1% agarose gel and was scored as homozygous susceptible (ASPCR band only with susceptible primers), homozygous resistant (ASPCR band only with *kdr* primers) or heterozygous (ASPCR band with both susceptible and *kdr* primers). Samples were always run alongside DNA from individuals with known genotypes determined by Sanger sequencing. At least one random sample from each population was also sequenced (see above) to validate the ASPCR results.

### Sequence analysis

Electropherograms were inspected with Chromas Lite (Technelysium Pty. Ltd., Tewantin, Queensland). We used the term haplotype to refer to the unique allele from sequencing each of the three (E6-8, E20-21 and E31-32) regions. Unknown haplotypes were identified by aligning sequences with known haplotypes using the MegAlign and EditSeq applications of Lasergene 7 (DNAStar, Madison, WI). Haplotypes present in heterozygous samples were identified either by cloning and sequencing (a minimum of two clones) or were inferred by comparison the known haplotypes in same populations on the electropherograms. All inferred haplotypes were observed in more than one individual.

We used the term “pattern sequences” to refer to specific concatenated sequences that were assembled from the sequence of E6-8, the codon from V410L (GTA for V, TTA for L), the sequence of E20-21, the codon from F1534C (TTC for F, TGC for C) and the sequence of E31-32 in each individual mosquito (Fig 1). Pattern sequences from heterozygous individuals were identified by comparison of known pattern sequences from the homozygous samples in the same population. Unique pattern sequences were numbered in the order they were discovered.

### Population structure analysis

STRUCTURE 2.3 [26] was used to infer the co-ancestry of individuals within the 24 populations from Asia and the Americas (Africa was not included because only a single population was available) using their pattern sequences. The STRUCTURE algorithm was run using a 100,000 burn-in period and 500,000 MCMC replications under the admixture model with correlated allele frequencies. This was repeated five times for each K, ranging from 1 to 5. The optimal number of K clusters was determined both following the guidelines of Pritchard et al [26] and the delta K method from Evanno et al [27] with the online version of STRUCTURE HARVESTER v.0.6.94 [28]. CLUMPP 1.12 [29] was used to aggregate results across the six independent STRUCTURE runs and provide the Q matrices based on which a population or an individual can be assigned to a specific cluster. The clustering results were visualized graphically using DISTRUCT v.1.1[30].

### Phylogenetic analyses

We used *Vssc* pattern sequences to create a phylogenetic tree based on maximum likelihood (ML). The program PhyML2.2.0 [31] plugin for Geneious Pro 10.2.2 [32] was used to perform maximum likelihood analyses with the substitution model (GTR+I+G), selected based on AIC in jModelTest 2.1.10 [33]. We assessed support for relationships on each phylogeny by performing 500 bootstrap replicates. In addition, we constructed the parsimony network to identify genealogic relations among patterns across sampling sites based on a TCS network in PopART [34,35]. The probability of parsimony is calculated for the number of mutational steps between patterns until the probability exceeds 0.95 in TCS network.

## Results

### Frequency and distribution of *kdr* alleles

**Fig 2:**
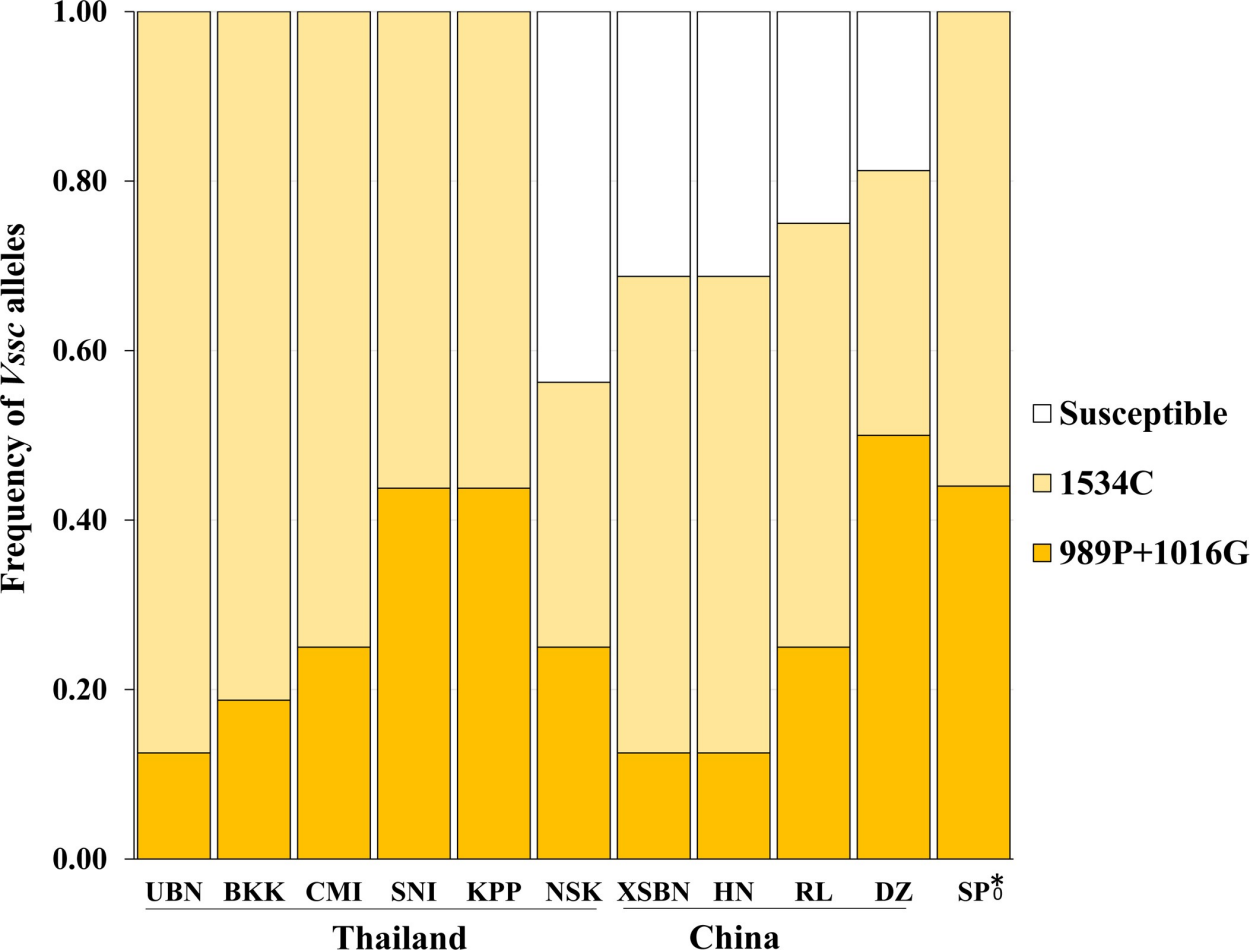
*Vssc* allele frequencies in populations of *A*. *aegypti* from Asia. Collections from Thailand included Ubon Ratchathani (UBN), Bangkok (BKK), Chiang Mai (CMI), Suratthani (SNI), Kamphaeng Phet (KPP), Nakhon Ratchasima (NSK). Strains from China included Xishuangbanna (XSBN), Hainan (HN), Ruili (RL) and Danzhi (DZ). * strain from Singapore (Kasai et al 2014).

**Fig 3:**
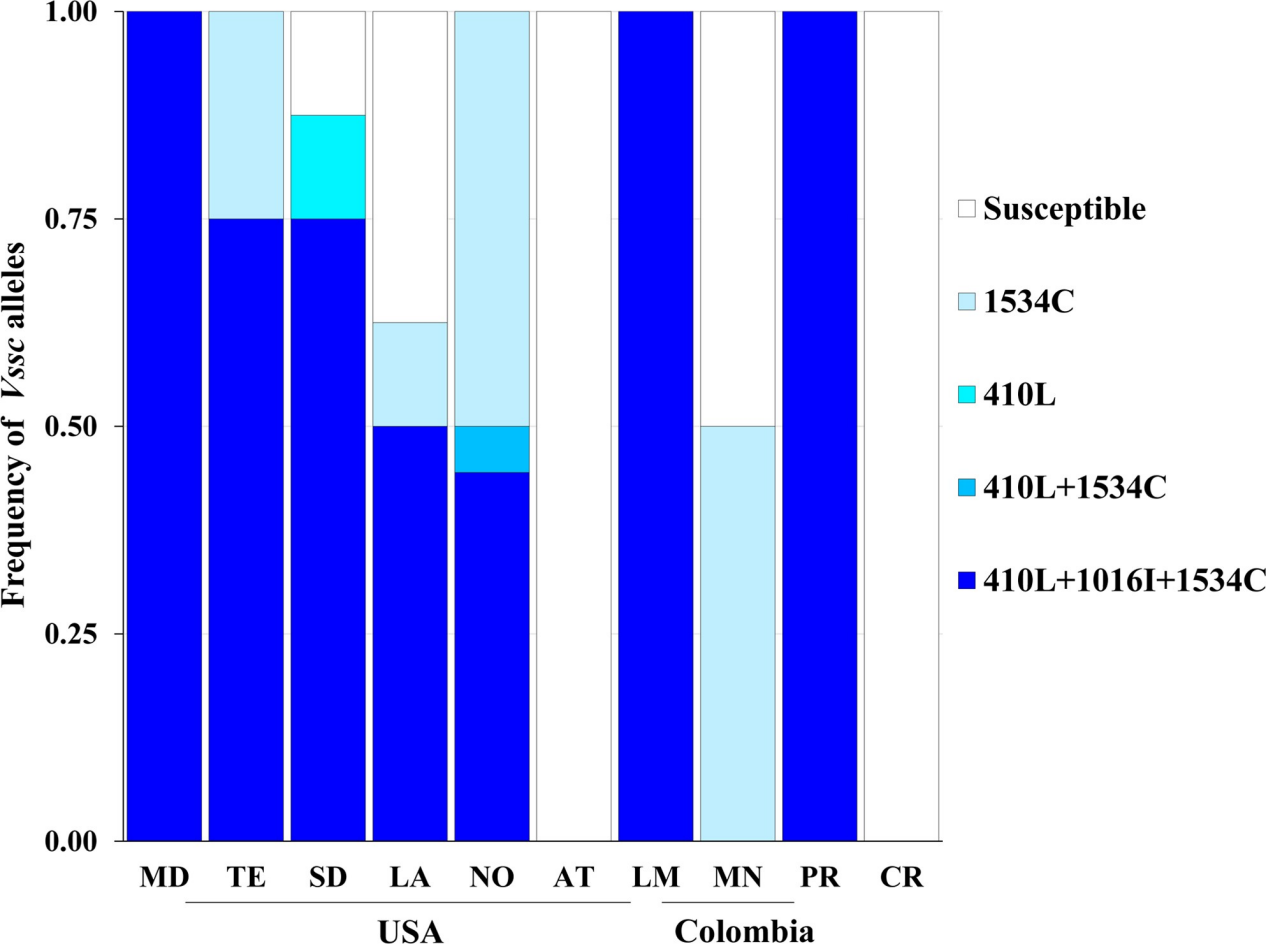
*Vssc* allele frequencies in populations of *A*. *aegypti* from the Americas. Collections from USA included Merced (MD), Tulare (TE), San Diego (SD), Los Angeles (LA), New Orleans (NO), St. Augustine (AT). Collections from Colombia included Acacias, Meta (MN), La Mesa (LM). Puerto Rico (PR) is from Puerto Rico. Costa Rica (CR) is from Costa Rica. RK (Cuba), SMK (USA) and OL (USA) are not included because they are susceptible strains with susceptible alleles and have been laboratory reared for many years.

There was considerable variation in the *Vssc* resistance alleles found in populations from Asia vs. the Americas. All Asian populations had only two types of resistance alleles: 1534C and 989P+1016G (Fig 2). The 1534C alleles were more common in each population, except Danzhou (DZ, China) with frequencies ranging from 0.31 to 0.88. Frequency of the 989P+1016G alleles ranged from 0.13 to 0.50. No susceptible alleles were found in the populations from Singapore [36] or from Thailand, except NSK. However, all Chinese populations contained susceptible alleles, with frequencies ranging from 0.19 (DZ) to 0.31(XSBN). Given that the 989P+1016G allele is known to confer pyrethroid resistance [37], all of these populations are resistant to pyrethroids. In populations from the Americas, four resistance alleles (410L, 410L+1534C, 410L+1016I+1534C and 1534C) were detected (Fig 3). Overall, the most common resistance allele was 410L+1016I+1534C, which was found in seven out of 10 populations, with allele frequencies ranging from 0.50 to 1.00. The second most common resistance allele was 1534C which was found in four out of 10 populations, with frequencies ranging from 0.13 to 0.50. The 410L allele was only detected in the SD population with a frequency of 0.13. The 410L+1534C allele was found only in the NO population at a frequency of 0.06. The resistant alleles 1534C and 410L+1016I+1534C were found in all populations from the USA, except AT. In the Colombian populations, the 410L+1016I+1534C allele was fixed in LM, whereas the MN had only the 1534C *kdr* allele (frequency of 0.50). No *Vssc* resistance alleles were detected in the AT and CR populations. The L982W, A1007G and I1011M/V mutations were not found in any mosquitoes, nor was the S989P+V1016G+F1534C allele which has been found in Myanmar [10], Saudi Arabia [18] and Malaysia [38]. All of the sequences have been deposited at GenBank (accession nos. MK977825-MK977851, S1 Table).

### Haplotype and population structure analysis

**Fig 4:**
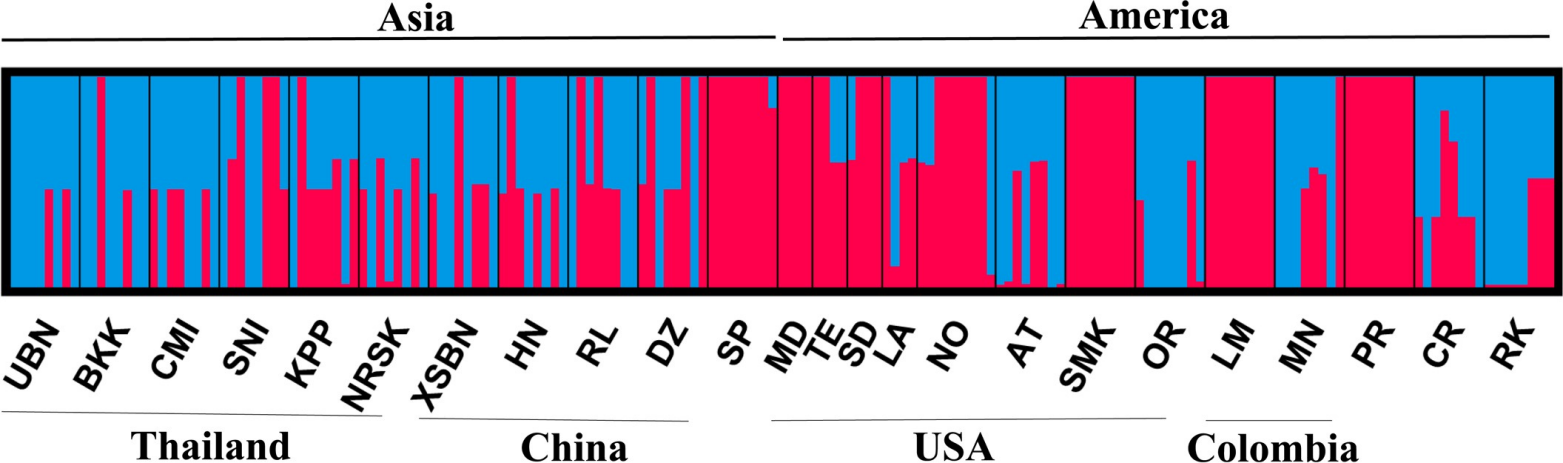
Population structure of *A*. *aegypti* populations from Asia and Americas used in this study. STRUCTURE bar plots indicate relatedness among geographic locations in 177 individuals. K = 2, and each cluster is indicated by different colors. Each vertical bar represents an individual. The height of the bars represents the probability of assignment to clusters.

Out of 192 individual mosquitoes examined, seven E6-8 haplotypes, nine E20-21 haplotypes, and eleven E31-32 haplotypes were identified (S1–S3 Figs). In E6-8 (~482 bp), exon 7 had one synonymous single nucleotide polymorphism (SNP), and there were 37 SNPs and 12 indels in introns 6 and 7. In E20-21 (~581 bp), exon 20 had 3 synonymous SNPs and 1 nonsynonymous SNP (S989P), exon 21 had 2 nonsynonymous substitutions (V1016I and V1016G), and intron 20 had 49 SNPs plus 20 indels. E20-21 haplotypes contained introns of 234 bp, except for v4, v8 and v9 which were 250 bp, and v1 which was 233 bp. Only one haplotype contained the 989P+1016G mutations (v8) and only one haplotype had the V1016I mutation (v9). The v4, v8 and v9 were identical in sequence except for the *kdr* mutations (S989P, V1016G/I). In the E31-32 region (1029 bp), exon 32 had 14 synonymous substitutions and 1 nonsynonymous substitutions (Q1835R), and intron 31 had 3 SNPs. The *Vssc* pattern sequences of 177 individuals from Asia and the Americas were used to analyze the population structure of these samples. It revealed admixture between the two groups despite the geographically distinct patterns for resistance mutations (Fig 4).

### Phylogenetic analyses

**Fig 5:**
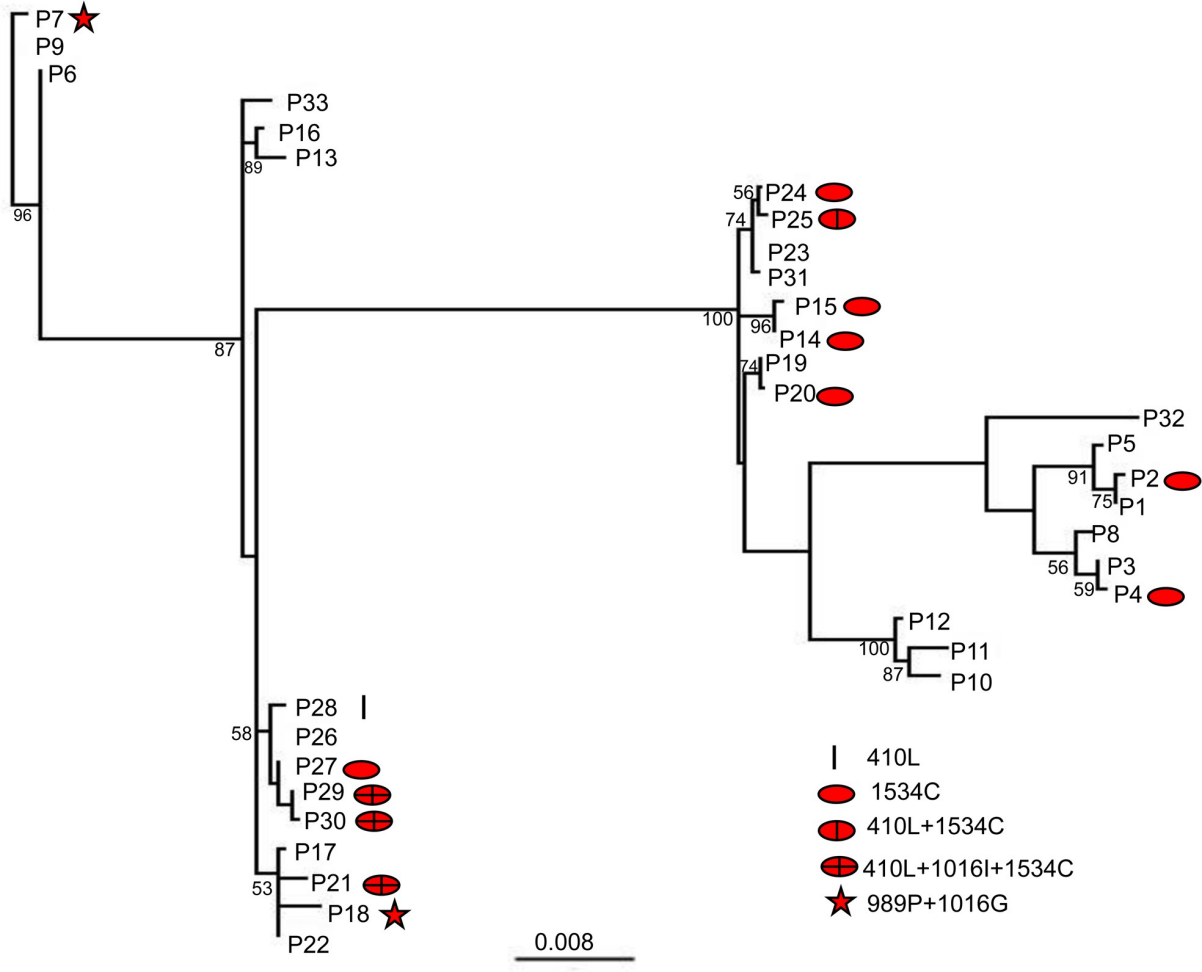
Maximum likelihood tree of the 33 pattern sequences found in *A*. *aegypti*. The pattern sequences are concatenated E6-8, V410L codon (GTA for V, TTA for L), E20-21, F1534C codon (TTC for F, TGC for C) and E31-32 in *Vssc*, and their types are represented by P number. ML bootstrap percentages are noted for clades ≥50%. The different *kdr* alleles are indicated by different symbols.

Phylogenetic analyses of 33 determined pattern sequences revealed multiple clades (with bootstrap support ≥90%)) (Fig 5). There are at least two independent clades containing 1534C alleles, suggesting multiple evolutionary origins of the F1534C mutation. Other resistance alleles were found in only a single pattern type (410L and 410L+1534C) or were found in two lineages (989P+1016G and 410L+1016I+1534C), making predictions of single or multiple evolutionary origins uncertain.

**Fig 6:**
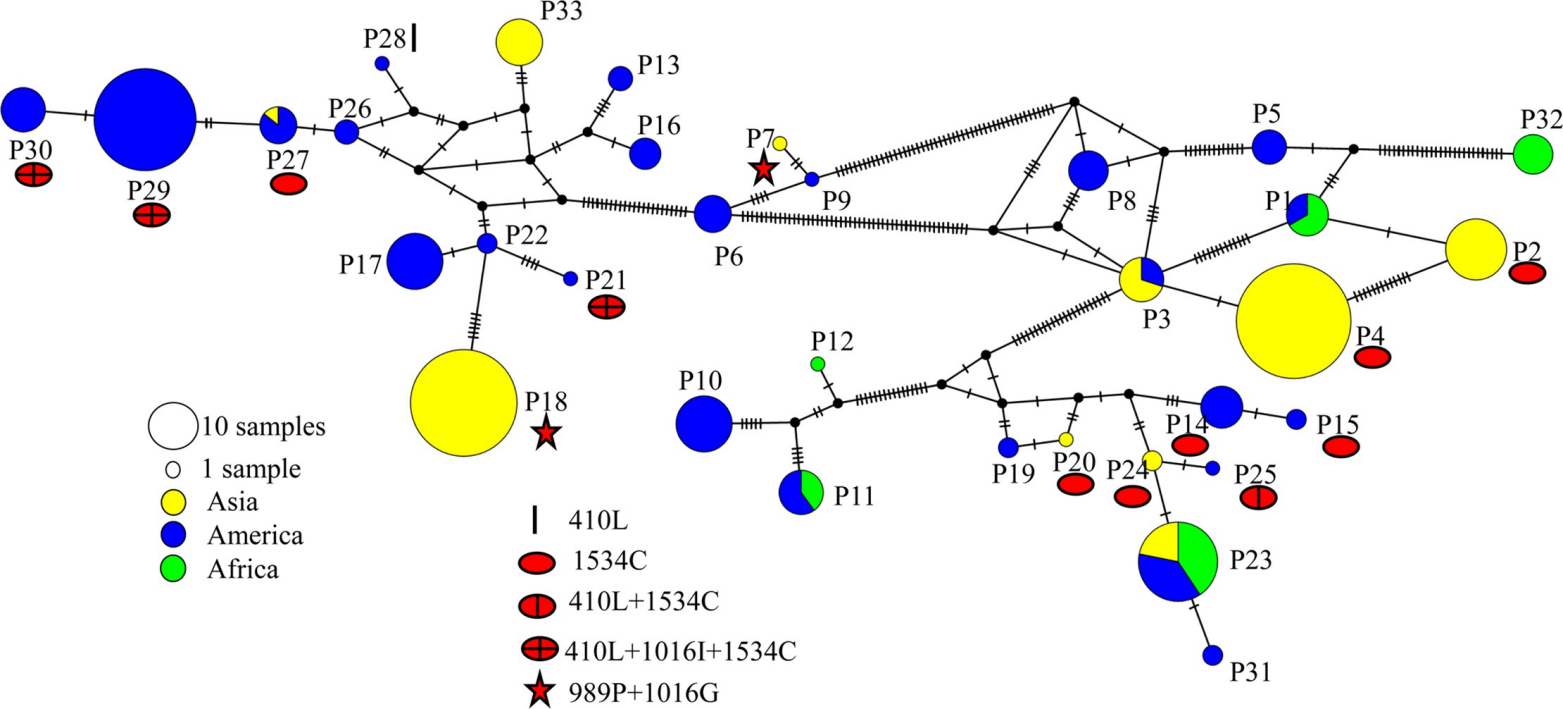
*Vssc* pattern sequences genealogy network constructed by TCS. The *Vssc* pattern sequences are concatenated E6-8, V410L codon (GTA for V, TTA for L), E20-21, F1534C codon (TTC for F, TGC for C) and E31-32) found in *A*. *aegypti*. The pattern sequences are connected to one another based on their similarity. Circles and numbers represent individual pattern sequences (S1 Table). The size of the circle represents the number of individuals having that pattern. Short hash-marks perpendicular to pattern branches indicate the number of base pair differences between pattern sequences. Black balls indicate intermediate missing (i.e. unsampled) steps. Geographic regions are coded by colors (Asia is yellow, America is blue and Africa is green). The different *kdr* alleles are indicated by different symbols.

The pattern sequences genealogy network illustrates frequency, relationships and number of mutations between the different pattern types (Fig 6). These results indicate pattern sequence P27 (1534C) and P28 (410L) were the result of one and two mutational steps, respectively, from their common susceptible ancestor P26. Similarly, P24 (1534C) and P25 (410L+1534C) are the results of one and two mutational step from P23, respectively. Also, the 410L pattern sequence (P28) and 410L+1534C pattern sequence (P25) were distributed in different lineages (Fig 5). These results suggest the 410L+1534C allele arose from in individual already having 1534C. The most common pattern sequences (P29 and P30) with 410L+1016I+1534C arose from P27 (1534C). However, neither the 410L+1534C nor 1016I+1534C were found in the lineage. The 410L+1016I+1534C pattern sequences and 410L+1534C pattern sequence were located in different lineages (Fig 5). This suggested the 410L+1016I+1534C allele more likely originated from a 1534C background by two additional mutations, but was not the result of additional one mutation (1016I) from a 410L+1534C background.

**Fig 7:**
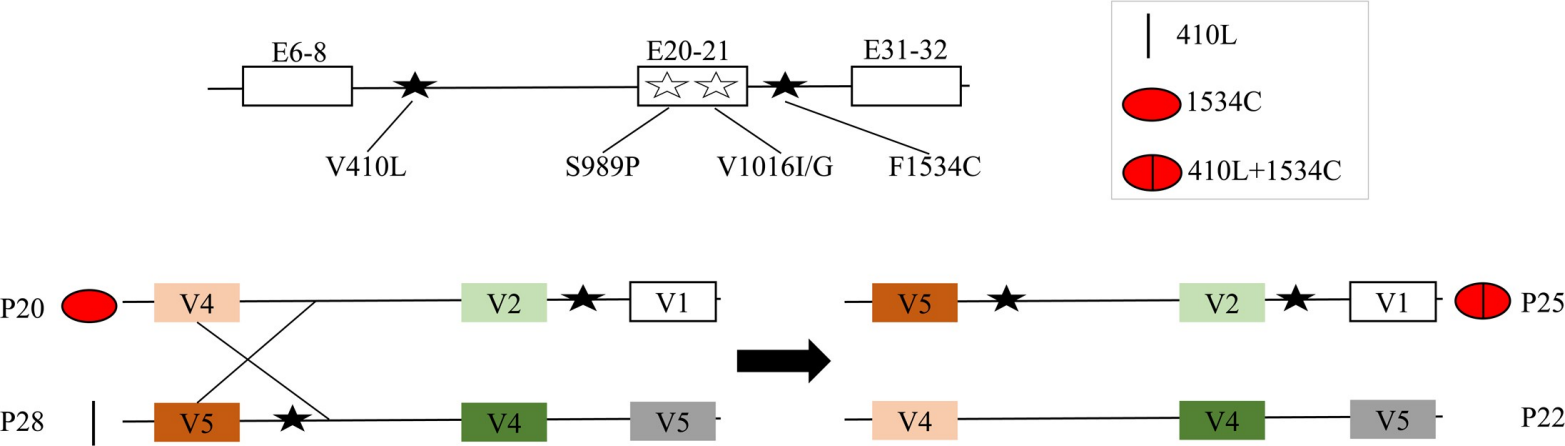
Recombination among the *kdr* pattern sequences in *A*. *aegypti*. The pattern sequences are concatenated E6-8, V410L codon (GTA for V, TTA for L), E20-21, F1534C codon (TTC for F, TGC for C) and E31-32 in *Vssc*.

In order to determine if recombination may contribute to the evolution of multiple mutations in *Vssc* alleles, we analyzed the observed pattern sequences associated with *kdr* mutations. We found sequences consistent with recombination events giving rise to the 410L+1534C allele (P25) from alleles having a 410L (P28) and a 1534C allele (P20) (Fig 7).

## Discussion

There are multiple *kdr* alleles in *A*. *aegypti*; some with global distribution and others that are found in more limited areas of the world (Fig 2 and Fig 3). The alleles containing only the F1534C mutation were widely distributed in the populations from Asia and the Americas. Alleles containing the S989P+V1016G mutations were found only in Asia and alleles with the V1016I mutation were only found in the Americas. These results are consistent with previous reports [2]. In 2017, the V410L mutation was detected in populations from Brazil [12]. It was subsequently found in *A*. *aegypti* from Colombia [13] and Mexico [14]. We also detected alleles with this mutation in mosquitoes from USA, Colombia and Puerto Rico (Fig 3). This suggests that *Vssc* alleles with the V410L mutation are widely distributed in *A*. *aegypti* from the Americas. We found a novel Q1835R mutation in the PR strain, but its association with the resistance requires further study.

The frequencies of the 989P+1016G and the 1534C alleles found in the Asian populations are intriguing. The S989P+V1016G allele confers pyrethroid resistance [36,37] and the F1534C allele has been shown to strongly correlate with pyrethroid resistance in *A*. *aegypti*. A field collected strain having both the 989P+1016G and 1534C alleles was selected with permethrin and the resulting strain had only the S989P+V1016G allele, suggesting that this allele gives greater protection to permethrin than the F1534C allele [4,36]. In all 11 of our Asian populations both the 1534C and 989P+1016G alleles were detected, and the 1534C allele was generally more common with frequencies ranging from 0.31 to 0.88 compared to 989P+1016G allele frequency that ranged from 0.13 to 0.50 (Fig 2). This suggests that the mosquitoes with the F1534C allele may have a lower fitness cost relative to the mosquitoes with the 989P+1016G allele. This finding is supported by Plernsub et al who reported that a strain having the F1534C mutation had no fitness reductions [39]. These results appear to be similar to the findings of *Vssc* alleles in house flies where *kdr-his* is common in some populations, even though it provides less resistance than other alleles [40–43].

Our results have both similarities and differences to what has previously been reported on *Vssc* alleles in the Americas. The populations we sampled from the USA had high frequencies of the 410L+1016I+1534C allele, and this is similar with Cornel *et al*. 2016 who reported that two populations from California were homozygous for V1016I mutation, although the 410 and 1534 positions were not examined [23]. Our mosquitos from Florida (AT) had no *kdr* alleles, and this is not consistent with Estep et al 2018 who found most *A*. *aegypti* strains from 62 locations in Florida were fixed or nearly fixed for the F1534C and V1016I mutations with frequencies from 0.11 to 1 [44]. Additional studies in Florida, across sites, seasons and years would be helpful to understand this discrepancy. In our populations from Colombia, the *kdr* allele types and frequencies are variable, and this is consistent with previous reports [13,45]. Generally, we detected four putative *kdr* alleles (410L, 410L+1534C, 410L+1016I+1534C and 1534C) in populations from Americas. The most common *kdr* allele was 410L+1016I+1534C. The PR strain was originally reported to have the 1016I+1534C allele [46], but the V410L position was not examined. Our results indicate that the PR population has the V410L+V1016I+F1534C allele. Thus, it is likely that the 1016I+1534C allele previously reported in *A*. *aegypti* is most likely the 410L+1016I+1534C allele. This also suggested that the high frequencies of 1016I or 1016I+1534C in previous studies are most likely high frequencies of 410L+1016I+1534C. This point is supported by Granada et al 2018 [13] and Saavedra-Rodriguez et al 2018 [14] in which the 1016I and 1016I +1534C alleles were rare, and the 410L+1016I+1534C allele was more common. Our primer sets of E20-21 are also capable of detecting previously reported mutations L982W, A1007G and I1011M/V[5,47], and none of the mutations were detected in any of the mosquitoes we analyzed, indicating they are rare or are geographically constrained.

It is well established that *A*. *aegypti* originated in Africa, invaded the Americas and later Asia from the Americas, likely in the 1890s [48–50]. Our population structure analysis showed that the populations from Asia and the Americas have similar background (Fig 4), although some *kdr* alleles have different geographic distributions (Fig 6). This supports the admixture of the populations from Asia and the Americas. For the population structure of mosquitoes from the Americas, it is important to consider that this species was eradicated from much of this area in the 1950s and 1960s, with re-colonization starting in the 1970s [49]. Therefore, it is possible that the mosquitoes with the V410L or V1016I mutations in populations from the Americas were from re-colonization by African populations. This idea is supported by previous reports [17,20] in which the V1016I (no S989P and V1016G) mutation was detected in African populations, unfortunately V410L was not examined in these two studies. Also, the 989P+1016G allele confers a >2000-fold resistance to DDT [37], so it is unlikely that the S989P+V1016G was ever present and then eradicated in American populations owing to the DTT use, and it is likely that the S989P and V1016G mutations occurred in Asian populations after the *A*. *aegypti* invaded Asia.

The intron between exons 20 and 21 is highly polymorphic in some species and being in close proximity to important *kdr* mutations has facilitated phylogenetic analyses of alleles in *Myzus persicae* [51], *Bemisia tabaci* [52], *Musca domestica* [53] and *Leptinotarsa decemlineata* [54]. While we did identify nine haplotypes from this region (E20-21) in *A*. *aegypti* (S2 Fig), this is far fewer than have been found for the species mentioned above. Thus, our ability to determine a single or multiple origin of the mutations (S989P, V1016G/I) in this region was unsuccessful. As additional E20-21 haplotype sequences become available, this analysis should be revisited.

A previous study on the E20-21 region of *Vssc* from *A*. *aegypti* identified two intron length polymorphisms: group A (250 bp) and B (234 bp) [55]. In our nine E20-21 haplotypes, introns of V4, V8 and V9 belong to intron group A, introns of V2, V3, V5, V6 and V7 belong to intron group B, and the intron of V1 differs from group B by the deletion of a single nucleotide (S2 Fig). The intron of group A was found to be strongly linked with S989P, V1016G/I and D1763Y mutations [6,56]. This agrees with our results in which the haplotypes containing S989P and V1016G/I mutations have the intron of group A (S2 Fig, V4, V8 and V9). Also, our results showed the F1534C mutation had no strong link to either the group A or group B. Specifically, the F1534C mutation was found with both intron A (V4-F1534C and V9-F1534C) and B (V2-F1534C and V1-F1534C), and they were found in populations from both Asia (V1-F1534C, V2-F1534C and V4-F1534C) and Americas (V2-F1534C, V4-F1534C and V9-F1534C) (i.e. showed no geographical distribution) (S1 Table). The findings are not consistent with previous reports in which the F1534C mutation was strongly linked with the intron of group A in Ghana [17] and intron of group B in Taiwan [17,56]. This is most likely that the two studies used *A*. *aegypti* populations from limited areas.

Previous studies on single vs. multiple evolutionary origins of *kdr* mutations have all suggested that multiple origins are more common [51–53,57]. Similarly, our results for F1534C suggest that this mutation had multiple evolutionary origins. However, our data could not differentiate between single and multiple evolutionary origins for the 989P+1016G and 410L+1016I+1534C allele. Additional sequences from other strains/populations will be needed before more concrete conclusions can be drawn.

**Fig 8:**
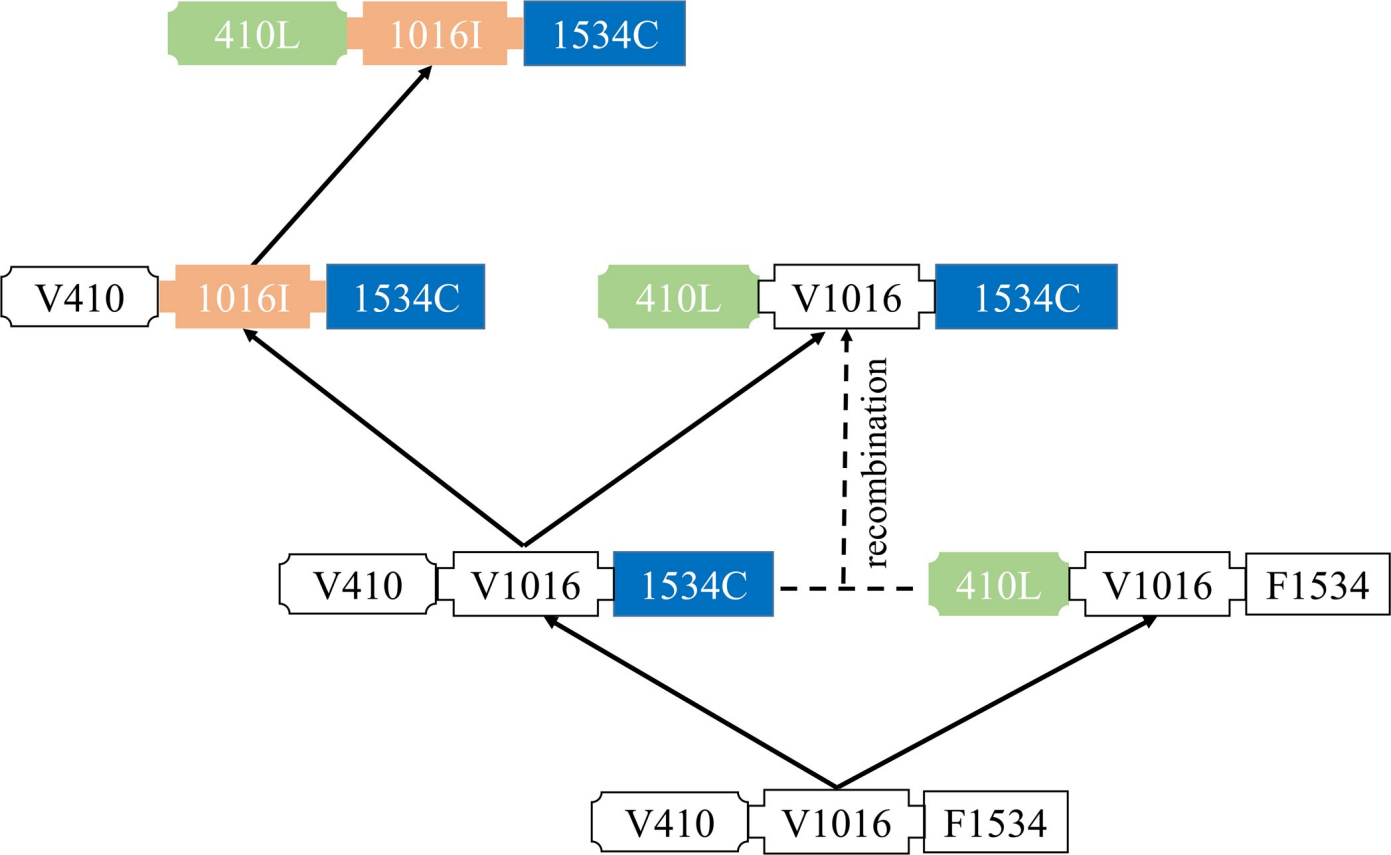
Proposed evolutionary scenarios of resistance alleles within the V410L, V1016I and 1534C mutations in *A*. *aegypti* populations. i) 410L+1534C occurs by addition of the 410L mutation to the 1534C allele, ii) 410L+1534C arises by crossover between the 410L and 1534C alleles, iii) 410L+1016I+1534C occurs sequential addition of the 1016I mutation and then the 410L mutation starting from the 1534C allele. Resistance alleles are indicated by different colors and box shapes.

Vera-Maloof et al [58] and Saavedra-Rodriguez et al [14] detected the frequencies of mutations in *A*. *aegypti* collected in Mexico from 2000–2012, and proposed the V410L, V1016I, and F1534C mutations arose independently, and then by two recombination events, gave rise to the 410L+1016I+1534C allele. In order to examine if alleles with multiple mutations arose by sequential accumulation of mutations, recombination or a combination of both processes, we analyzed our 33 pattern sequences. Our data equally support the ideas that the 410L+1534C allele could have arisen by recombination (Fig 7) or by addition of the 410L mutation to an individual having the F1534 allele (Fig 6). Without accurate knowledge of mutation and recombination rates it is difficult to decide which model is most likely. Our data suggests that other *kdr* alleles having multiple mutations could have arisen by sequential accumulation of mutations, but we found no evidence to support that they arose via recombination. However, a larger dataset of alleles would be needed before solid conclusions can be made. Although no 1016I+1534C allele was found in our samples, it has been reported in mosquitoes from Colombia [13] and Mexico[14]. Therefore, based on the sequence polymorphisms associated with the mutations, we put forward possible evolutionary scenarios of how resistance alleles with multiple mutations may have evolved (Fig 8). It appears the 410L+1534C allele occured by addition of a V410L mutation to an individual already having the F1534C allele or by recombination (see above), ii) the 410L+1016I+1534C allele arose in an individual having the 1016I +1534C allele which originates from an individual with a1534C allele. It should be mentioned that we analyzed the relationship between the pattern sequences, not considering geographyical distribution of alleles (because our analysis focused on identification of the most common alleles in each population, not on a deep sampling). Although the 989P+1016G+1534C allele has been reported in *A*. *aegypti* populations [18,19,38], it was not detected in our samples. Therefore we cannot propose how this allele arose.

In summary, we examined nine *Vssc* mutations (V410L, L982W, S989P, A1007G, I1011V/M, V1016G/I and F1534C) and 33 pattern sequences of individual *A*. *aegypti* from 25 geographic collections. Our results show that 1534C and 989P+1016G alleles are widely distributed in Asian populations. The distinct 410L, 410L+1534C, 410L+1016I+1534C and 1534C alleles were found in populations from the Americas where the most common *kdr* allele was 410L+1016I+1534C, followed by 1534C. The phylogenetic analysis of *Vssc* suggest multiple independent origins of the 1534C allele. Our data suggest the 410L+1534C allele could have arisen by accumulation of the V410L mutation in an individual already having the 1534C allele or by the crossover of the 410L and 1534C alleles. More *kdr* pattern sequences (i.e. 1016I+1534C and 989P+1016G+1534C) would allow for understanding of how multiple mutations in *Vssc* have arisen and evolved. Overall, our data corroborate previous geographic distributions of *kdr* mutations and provides evidence for both recombination and sequential accumulation of mutations contributing to the molecular evolution of *kdr* mutations in *A*. *aegypti*.

## Supporting information

S1 Table Pattern sequences concatenated E6-8, V410L codon (GTA for V, TTA for L), E20-21, F1534C codon (TTC for F, TGC for C) and E31-32 in *Vssc* in *A*. *aegypti*. (XLSX)

S1 Fig The sequence alignment of haplotypes of E6-8 in *Vssc* of *A*. *aegypti*. The sequences were determined by direct sequencing of PCR products using reverse primers. The introns are shaded and bases differing from the majority are red.
(DOCX)

S2 Fig The sequence alignment of haplotypes in E20-21 in *Vssc* of *A*. *aegypti*. The sequences were determined by direct sequencing of PCR products using reverse primer. The introns are shaded and bases differing from the majority are red. # S989P mutation (TCC for S989, CCC for 989P), * and & V1016I and V1016G mutations (GTA for V1016, ATA for V1016I, GGA for 1016G), respectively. The introns of V4, V8 and V9 belong to haplotype group A, others belong to haplotype group A according to Martins et al. 2009 [55].
(DOCX)

S3 Fig The sequence alignment of haplotypes in E30-31 in *Vssc* of *A*. *aegypti*. The sequences were determined by direct sequencing of PCR products using reverse and forward primers. The intron is shaded and bases differing from the majority are red.
(DOCX)
