# Supplementary material for: Evidence for both sequential mutations and recombination in the evolution of kdr alleles in Aedes aegypti
Source: PLoS Negl Trop Dis. 2020 Apr 17;14(4):e0008154. doi: 10.1371/journal.pntd.0008154 (PMC7164583; doi:10.1371/journal.pntd.0008154)
Supplement: S2 Fig — The sequences were determined by direct sequencing of PCR products using reverse primer. The introns are shaded and bases differing from the majority are red. # S989P mutation (TCC for S989, CCC for 989P), * and & V1016I and V1016G mutations (GTA for V1016, ATA for V1016I, GGA for 1016G), respectively. The introns of V4, V8 and V9 belong to haplotype group A, others belong to haplotype group A according to Martins et al. 2009 [55]. (DOCX) [file pntd.0008154.s003.docx]

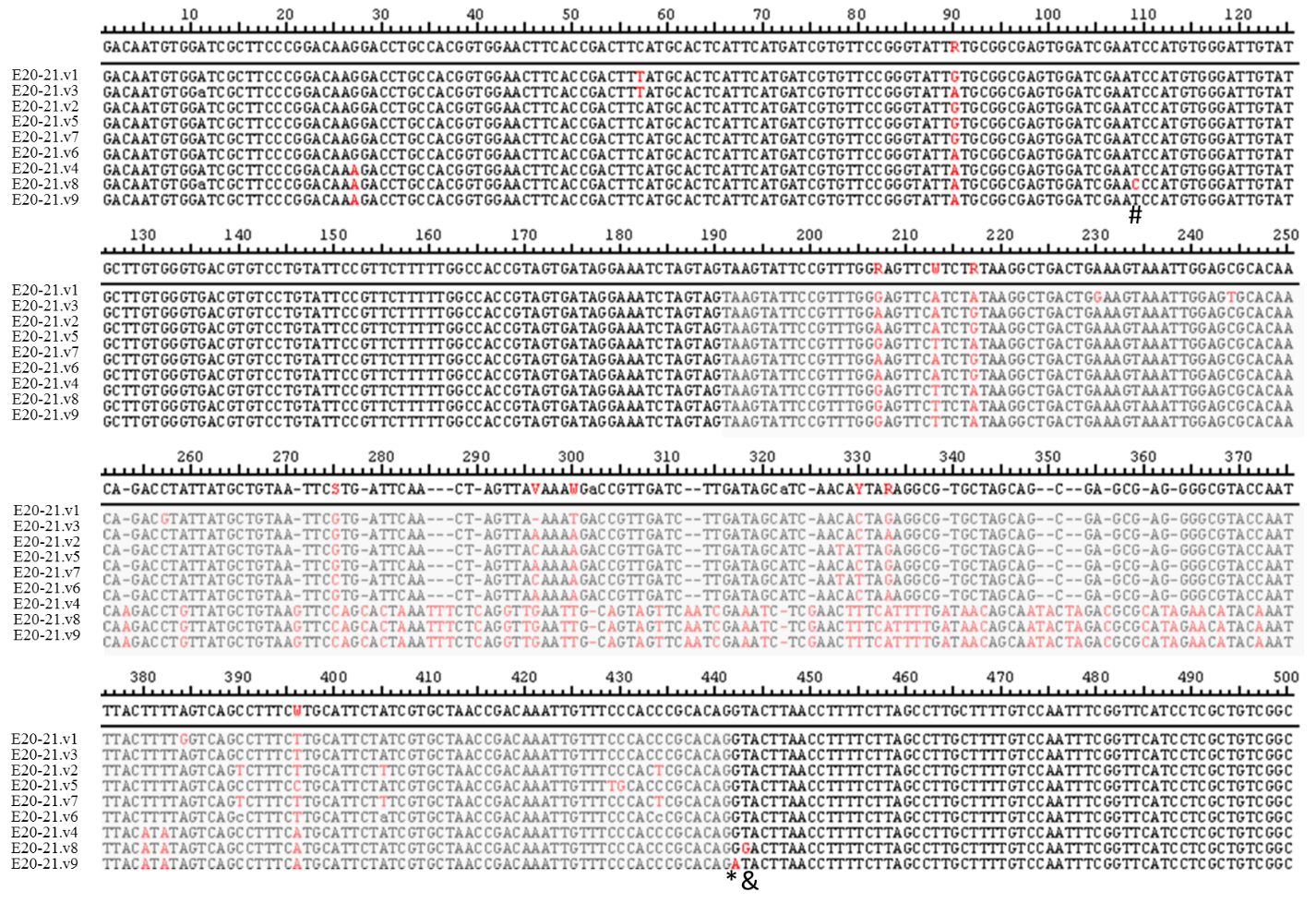


**S2 Fig. The sequence alignment of haplotypes in E20-21 in Vssc of *A. aegypti***. The sequences were determined by direct sequencing of PCR products using reverse primer. The introns are shaded and bases differing from the majority are red. The introns of V4, V8 and V9 belong to haplotype group A, others belong to haplotype group A according to Martins et al. 2009.

# S989P mutation (TCC for S989, CCC for 989P)

* V1016I mutation (GTA for V1016, ATA for V1016I).

& V1016G mutation (GTA for V1016, GGA for 1016G).
