# Supplementary material for: Evidence for both sequential mutations and recombination in the evolution of kdr alleles in Aedes aegypti
Source: PLoS Negl Trop Dis. 2020 Apr 17;14(4):e0008154. doi: 10.1371/journal.pntd.0008154 (PMC7164583; doi:10.1371/journal.pntd.0008154)
Supplement: S3 Fig — The sequences were determined by direct sequencing of PCR products using reverse and forward primers. The intron is shaded and bases differing from the majority are red. (DOCX) [file pntd.0008154.s004.docx]

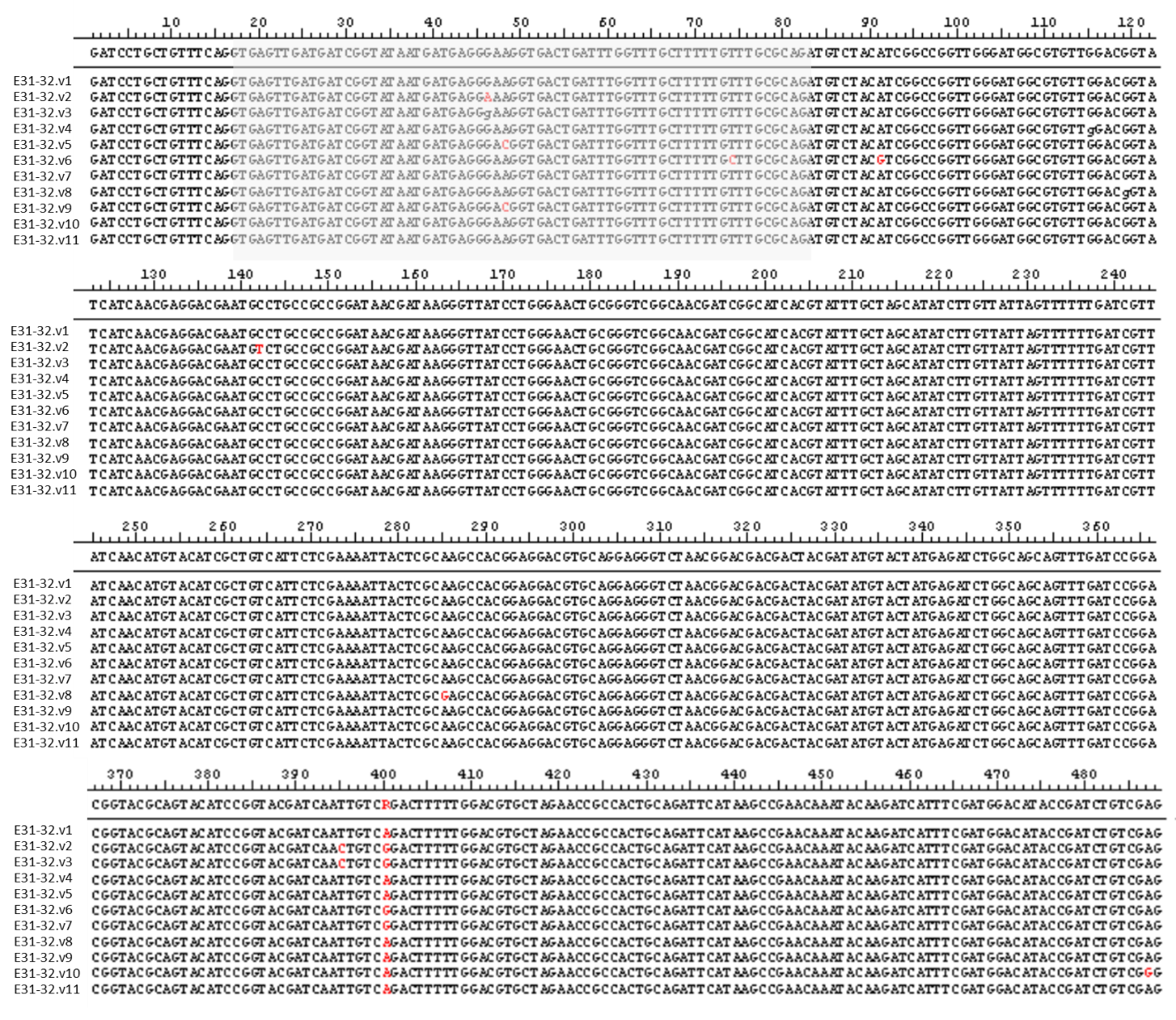


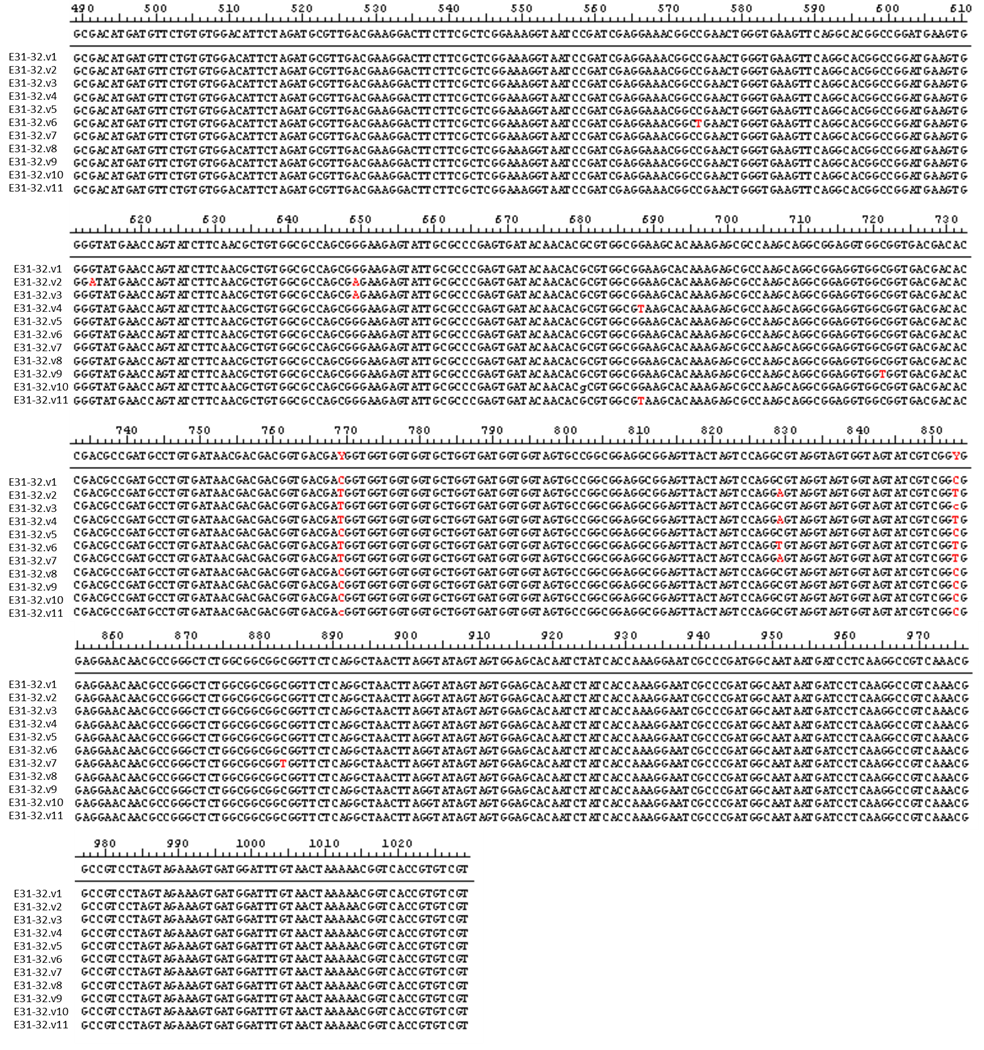


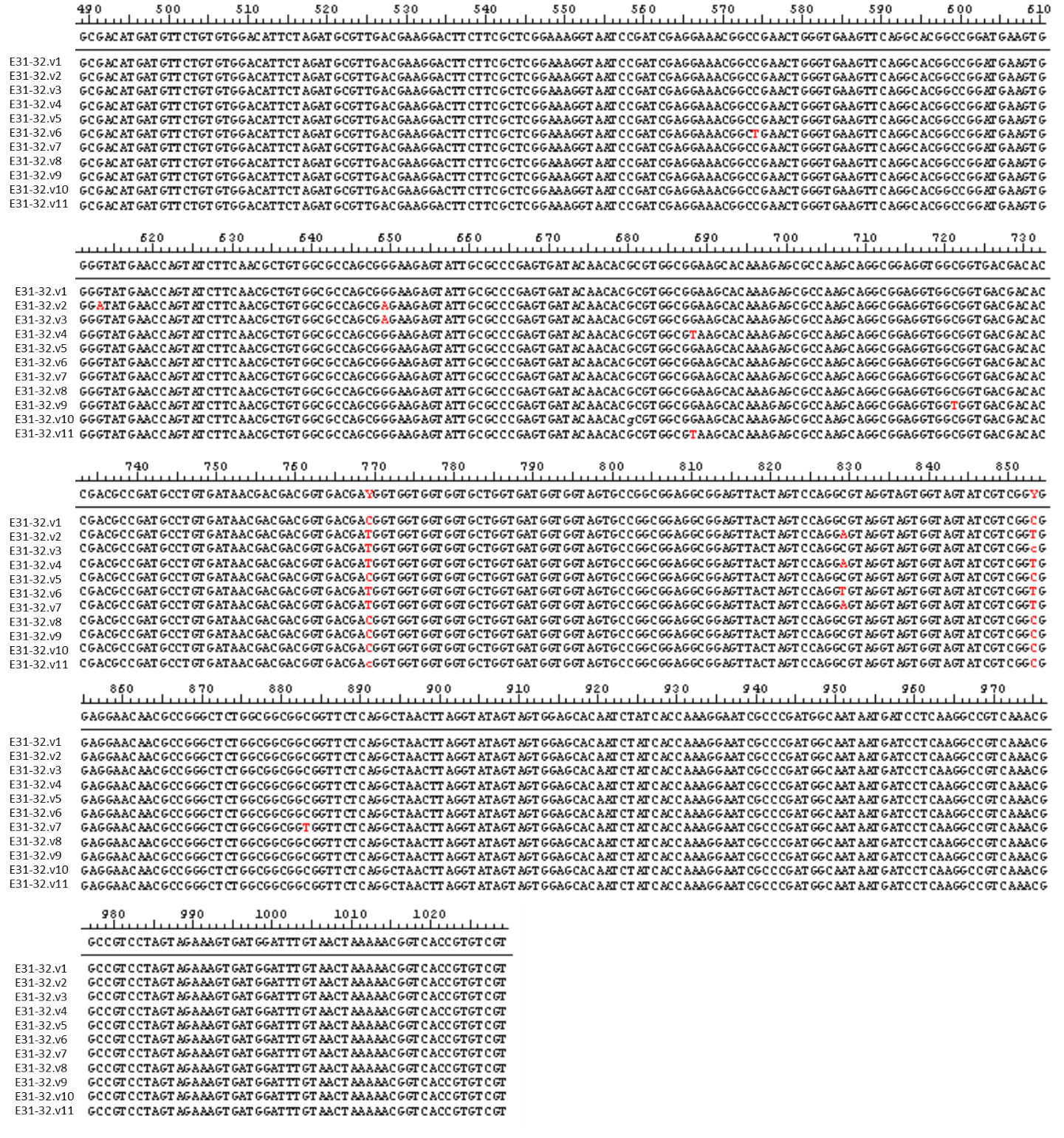


**S3 Fig. The sequence alignment of haplotypes in E30-31 in *Vssc* of *A. aegypti***. The sequences were determined by direct sequencing of PCR products using reverse and forward primers. The intron is shaded and bases differing from the majority are red.
